# NKAP Regulates Senescence and Cell Death Pathways in Hematopoietic Progenitors

**DOI:** 10.3389/fcell.2019.00214

**Published:** 2019-10-02

**Authors:** Michael Jeremy Shapiro, Joshua Anderson, Michael Jonathan Lehrke, Meibo Chen, Molly Nelson Holte, Virginia Smith Shapiro

**Affiliations:** Department of Immunology, Mayo Clinic, Rochester, MN, United States

**Keywords:** NKAP, hematopoiesis, cyclin dependent kinase inhibitor, senescence, apoptosis

## Abstract

NKAP is a multi-functional nuclear protein that has been shown to be essential for hematopoiesis. Deletion of NKAP in hematopoietic stem cells (HSCs) was previously found to result in rapid lethality and hematopoietic failure. NKAP deficient cells also exhibited diminished proliferation and increased expression of the cyclin dependent kinase inhibitors (CDKIs) p19 Ink4d and p21 Cip1. To determine how dysregulation of CDKI expression contributes to the effects of NKAP deficiency, NKAP was deleted in mice also deficient in p19 Ink4d or p21 Cip1 using poly-IC treatment to induce Mx1-cre. Hematopoietic failure and lethality were not prevented by deficiency in either CDKI when NKAP was deleted. Inducible deletion of NKAP in cultured hematopoietic progenitors *ex vivo* resulted in a senescent phenotype and altered expression of numerous cell cycle regulators including the CDKI p16 INK4a. Interestingly, while combined deficiency in p16 INK4a and p21 Cip1 did not reverse the effect of NKAP deficiency on hematopoiesis *in vivo*, it did shift the consequence of NKAP deficiency from senescence to apoptosis in *ex vivo* cultures. These results suggest that NKAP may limit cellular stress that can trigger cell cycle withdrawal or cell death, a role critical for the maintenance of a viable pool of hematopoietic progenitors.

## Introduction

Hematopoiesis begins with a small population of HSCs in the bone marrow (Sigvardsson, 2009; Matsumoto and Nakayama, 2013). Infrequent cell divisions both renew the HSC population and allow differentiation into more proliferative, though uncommitted, types of progenitor cells such as short term HSCs and multipotent progenitors. Differentiation along with additional proliferation ultimately produces the full range of hematopoietic lineages. Sustained hematopoiesis over the life of an organism requires an appropriate balance between proliferation and differentiation. Studies of mice with mutations in cell cycle regulators have revealed that disruption of this balance can result in stem cell exhaustion and hematopoietic failure (Sigvardsson, 2009; Matsumoto and Nakayama, 2013). NKAP is a multifunctional nuclear protein that can act as a transcriptional repressor and associate with a component of the Notch corepressor complex and with HDAC3, which mediates epigenetic modifications associated with gene silencing (Pajerowski et al., 2009). NKAP has also been shown to contribute to splicing and RNA processing (Burgute et al., 2014; Fica et al., 2019; Zhang et al., 2019) and to chromosome alignment during mitosis (Li et al., 2016). Lineage specific deletion of the NKAP gene has revealed its role in multiple cell types. It is required for development and maturation of conventional T cells, iNKT cells, and Tregs (Pajerowski et al., 2009; Hsu et al., 2011; Thapa et al., 2013, 2016; Dash et al., 2018), and is also essential for hematopoiesis (Pajerowski et al., 2010). Deletion of NKAP in the HSC lineage using Vav-cre resulted in perinatal lethality, depletion of all hematopoietic lineages, and the absence of a detectable HSC population in the bone marrow (Pajerowski et al., 2010). Deletion of NKAP in adult cKO mice using an inducible Mx1-cre transgene resulted in hematopoietic failure and rapid lethality. Critically, this phenotype was shown to be cell intrinsic as it was recapitulated after hematopoiesis was reconstituted in irradiated WT recipients using bone marrow from Mx-1-Cre NKAP cKO mice (Pajerowski et al., 2010).

NKAP deficient HSCs displayed decreased proliferation and dysregulation of two CDKIs, which act to slow or arrest cell cycle progression. Specifically, elevated expression of p21 Cip1 (also known as p21 Waf1) and p19 Ink4d was observed (Pajerowski et al., 2010). p21 Cip1 deficient hematopoietic progenitor cells have a diminished ability to reconstitute hematopoiesis in transplantation experiments and are hyperproliferative *in vitro* (Stier et al., 2003). Thus, p21 Cip1 is believed to restrict the proliferation of HSCs and prevents stem cell exhaustion (Cheng et al., 2000). As NKAP was also found to bind to the p21 Cip1 promoter by chromatin immunoprecipitation (Pajerowski et al., 2010), it seemed possible that loss of NKAP in HSCs might result in defects in hematopoiesis specifically due to dysregulation of the pathways controlling CDKI expression. Here, we show that additional deficiency in p21 Cip1 or p19 Ink4d does not abrogate the effect of NKAP deficiency in hematopoiesis. Deletion of NKAP in hematopoietic progenitors analyzed *ex vivo* did in fact result in increased expression of multiple CDKI genes and other changes indicative of a senescent phenotype. However, loss of NKAP in cells also deficient in both p21 Cip1 and p16 Ink4a resulted in the rapid onset of apoptosis instead of senescence, suggesting that loss of NKAP leads to cellular stress incompatible with survival in proliferating cells.

## Materials and Methods

### Mice

Mx1-cre NKAP cKO mice were described previously (Pajerowski et al., 2010). Littermates either lacking Mx1-cre or in which the NKAP gene was not floxed were utilized as WT controls for *in vivo* experiments. Mice with an ER-cre transgene were obtained from Jackson labs, and these were crossed to produce ER-cre NKAP cKO mice. p16 Ink4a KO mice (Sharpless et al., 2001) were from NCI Frederick, p19 Ink4d KO mice (Zindy et al., 2000) were from MMRRC, and p21 Cip1 KO mice (Deng et al., 1995) were from Jackson labs. For simplicity, these mice will be referred to, respectively, as “p16 KO,” “p19 KO,” “p21 KO,” and “p16/p21 dKO,” followed by “Mx1-Cre NKAP cKO” or “Er-cre NKAP cKO” where applicable. All animal studies were carried out in accordance with and with approval from the Mayo Clinic Institutional Animal Care and Use Committee.

### In vivo Deletion of NKAP

Mice were treated with poly-IC to induce Mx1-cre and monitored for up to 20 days as previously described (Pajerowski et al., 2010). Mice were examined daily and lethality recorded if the mice were found dead or required euthanasia due to severe morbidity. Surviving mice were euthanized at day 20. Peripheral blood was collected upon euthanasia and complete blood counts obtained as described (Pajerowski et al., 2010). To examine bone marrow cellularity, femurs were fixed in formalin and paraffin embedded sections were then generated and stained with hematoxylin and eosin.

### Hematopoietic Progenitor Cultures

Cultures were derived from ER-cre NKAP cKO mice or WT controls with a floxed NKAP gene but lacking ER-cre. Bone marrow was flushed from femurs from individual mice using PBS and filtered through a cell strainer. Cells were collected by centrifugation and resuspended at 100 million/ml in separation buffer (Ca/Mg free PBS with 0.5% BSA and 2 mM EDTA). The following biotinylated antibodies (Tonbo) were added to 1 μg/ml: CD3e (clone 2C11), Ly76 (clone Ter119), CD11b (clone M1/70), CD19 (clone 1D3), CD45R (clone B220), Ly6G (clone GR1). Cell suspensions were then incubated for 20 min at 4°C, washed twice, and resuspended in 0.5 ml separation buffer. 25 μl of Streptavidin MicroBeads (Miltenyi Biotec) was added and the suspensions rotated for 20 min at 4°C and then washed twice. Cells were applied to an LD column attached to a QuadroMACS (Miltenyi Biotec) followed by 2 ml of separation buffer. The cells not retained on the column were collected. Cells were then cultured at 37°C in IMDM media supplemented with 10% FCS, 2 mM Glutamine, 100 U/ml antibiotic mixture, 55 μM βME, 100 ng/ml Flt ligand, 20 ng/ml SCF, 20 ng/ml IL6, and 20 ng/ml TPO (all cytokines from PeproTech). Cells were initially cultured in a single well of a 6 well dish and split into multiple wells as necessary over a 7 to 10 day period. One to four days prior to analysis, 4-hydroxytamoxifen was added to the cultures at a final concentration of 0.1 μM to trigger NKAP deletion. Cell numbers were monitored using a Countess automated counter (Invitrogen).

### Analysis of Gene Expression by RT-PCR

RNA was isolated using an RNeasy Mini kit (Qiagen) and cDNA was then generated from equal quantities of RNA from each condition using the Superscript III system (Invitrogen). QPCR reactions were set up using Universal Taqman Master Mix and Taqman gene expression assays purchased from Invitrogen and run on a Step One Plus instrument (Applied Biosystems). cDNA was generated from cultures initiated from three separate pairs of mice, each cDNA was analyzed in triplicate PCR reactions, and average relative gene expression for each condition calculated using the 2^–ΔΔC_T_^ method (Livak and Schmittgen, 2001). Taqman assays were as follows: NKAP (Mm00482418), p21/cdkn1a (Mm04205640), p27/cdkn1b (Mm00438168), p57/cdkn1c (Mm004386170), p16/cdkn2a (Mm00494449), p19/cdkn2d (Mm00486943), IL1B (Mm00434228), IL6 (Mm00446190), CXCL1 (Mm04207460), CSF2 (Mm01290062), and Myc (Mm00487804). 18S rRNA (4352930) was used for normalization.

### Western Blot Analysis of Cell Cycle Progression and Apoptosis

Cell lysates were generated by resuspending cells at equal concentrations directly in sample buffer. Lysates were sheared with a 28 g needle and boiled before loading on a gel. Western blotting was then performed using the following antibodies: phosphorylated Histone 3(S10) (Cell Signaling #CST3377), PCNA (Cell Signaling #CST2586), CDK1 (Abcam #ab32384), CDK2 (Abcam #ab32147), actin (Sigma #A3584), Ubiquityl-Histone H2A(K119) (Cell Signaling #CST8240), PARP (Cell Signaling #CST9532), and Cleaved PARP (Cell Signaling #CST94885). Equivalent results were obtained from samples taken from cultures initiated from pairs of mice on three separate occasions and representative data is shown.

### Analysis of Apoptosis

Cultured cells were analyzed using an Annexin V FITC apoptosis detection kit (eBioscience), FVD Ghost Dye v450 (Tonbo Biosciences) and an antibody to cleaved caspase-3 (Cell Signaling #CST9602) according to the manufacturer’s instructions.

### Statistics

In all figures, error bars represent standard deviation. *P*-values were determined by unpaired Student’s *t*-test using Microsoft Excel.

## Results

**FIGURE 1:**
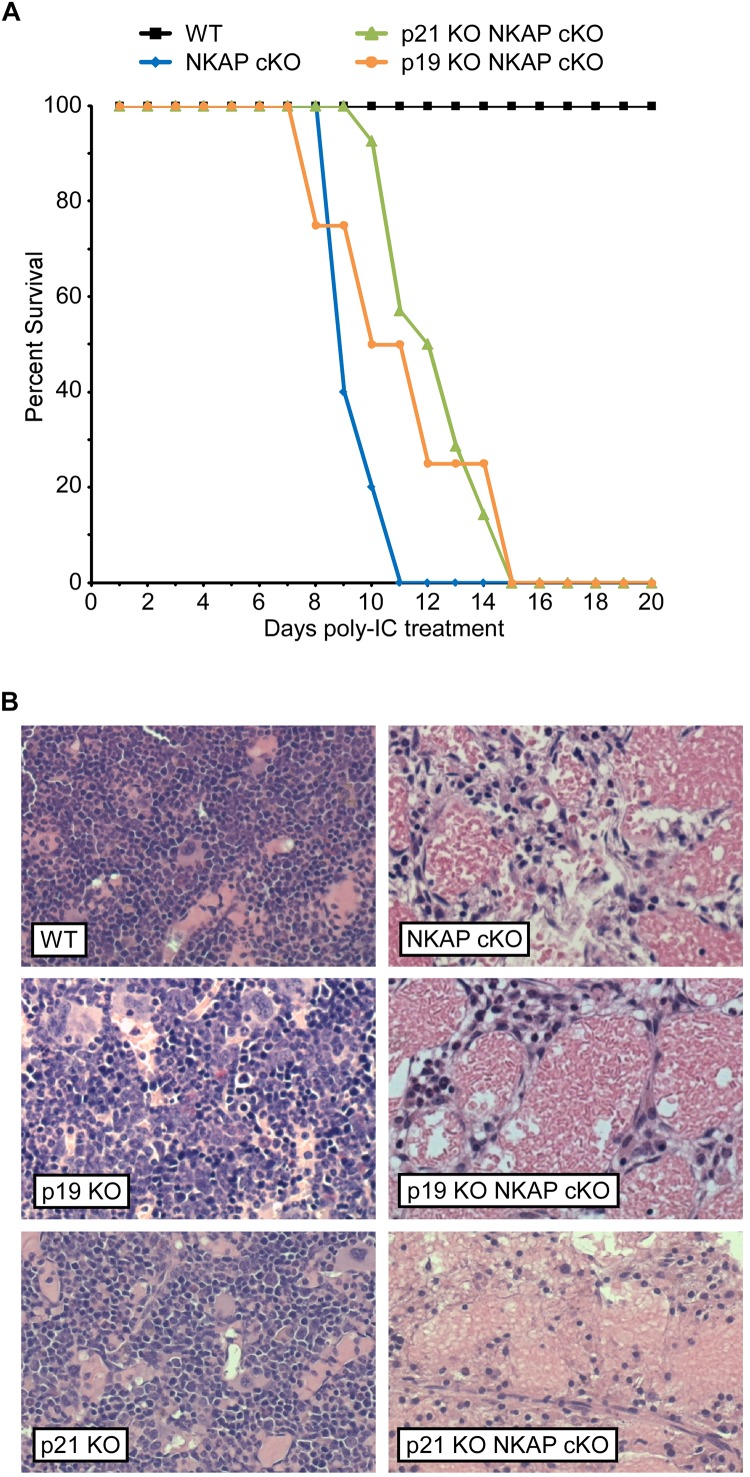
NKAP deficiency results in lethality and hematopoietic failure. **(A)** Survival was examined in 3 WT, 7 Mx1-cre NKAP cKO, 14 p21 KO Mx1-cre NKAP cKO, and 4 p19 KO Mx1-cre NKAP cKO mice (see section “Materials and Methods” for nomenclature). All mice were treated with poly-IC and monitored daily for up to 20 days. Lethality was recorded if the mice were found dead or required euthanasia due to severe morbidity. The percent of mice in each group surviving to the day indicated by the horizontal axis is shown on the vertical axis. **(B)** Femurs from poly-IC treated mice of the indicated genotypes were fixed in formalin at the end of the time course and paraffin embedded sections were then generated and stained with hematoxylin and eosin. Brightfield images were obtained at 20× magnification (Plan Fluotar 0.4 aperture lens) at room temperature without immersion medium using a Leica DMI300B microscope and EC3 color camera. Leica Application Suite software was used to obtain the images and Canvas software used to prepare the figure. Representative images are shown.

To determine if upregulation of either p19 Ink4d or p21 Cip1 was the direct cause of the hematopoietic failure previously observed to result from NKAP deficiency (Pajerowski et al., 2010), deletion of NKAP was induced in mice also deficient in these genes (see section “Materials and Methods” for mouse nomenclature). As described previously, Mx1-cre induction upon poly-IC treatment resulted in hematopoietic failure and the death of all Mx1-cre NKAP cKO mice examined within 20 days (Figure 1A) along with hypocellular bone marrow (Figure 1B). In contrast, poly-IC treatment did not result in lethality, hypocellular bone marrow, or any apparent distress in WT mice. In both p19 KO Mx1-cre NKAP cKO and p21 KO Mx1-cre NKAP cKO mice, poly-IC treatment resulted in hypocellular bone marrow and lethality within a similar time period as observed in Mx1-cre NKAP cKO mice. Therefore, the effect of NKAP deficiency on hematopoiesis cannot be solely attributed to dysregulation of p19 Ink4d or p21 Cip1 gene expression.

**FIGURE 2:**
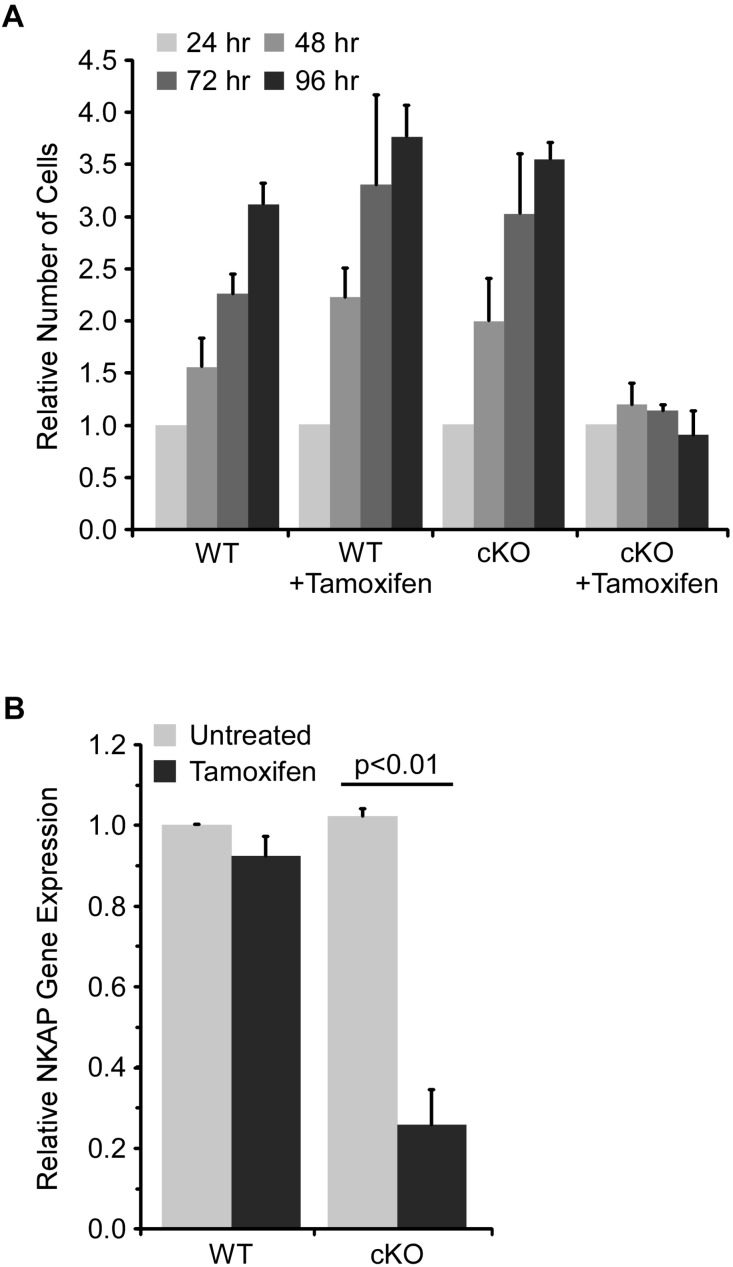
NKAP deficiency impairs expansion of hematopoietic progenitor cultures. Hematopoietic progenitor cells were cultured *ex vivo* and treated with tamoxifen to induce NKAP deletion. Cells were either from mice with a floxed NKAP gene (WT) or floxed NKAP along with an ER-cre transgene (cKO). **(A)** The indicated cultures were treated with tamoxifen to induce NKAP deletion, or left untreated, and the number of cells in each group counted daily for 4 days. The number of cells at each time point relative to the number at 24 h is shown. The data was averaged by monitoring cultures initiated from three separate pairs of mice. **(B)** RNA was obtained from cells after 4 days of tamoxifen treatment and expression of NKAP was determined by RT-PCR using a Taqman gene expression assay. The data shown is average gene expression determined from four independent experiments initiated from separate pairs of mice, and normalized to the untreated WT group.

The requirement for NKAP in early hematopoiesis was investigated further using an *ex vivo* model. Specifically, a mixture of hematopoietic progenitor cells was isolated from bone marrow and then cultured for up to ten days with cytokines to support survival and proliferation. ER-cre NKAP cKO cells expressed an ER-cre fusion protein which, upon activation by tamoxifen, will delete the floxed NKAP gene. Cells (designated WT) with floxed NKAP but lacking ER-cre were used as controls. As shown in Figure 2A, the number of cells in the WT cultures increased over time and there was no effect of tamoxifen treatment. Untreated ER-cre NKAP cKO cultures also expanded at a similar rate. However, the tamoxifen treated ER-cre NKAP cKO cells remained at an approximately constant number over a four day time course. Expression of NKAP was also found to be significantly reduced in the tamoxifen treated cKO cells relative to the other conditions, confirming that deletion of the gene occurred (Figure 2B).

**FIGURE 3:**
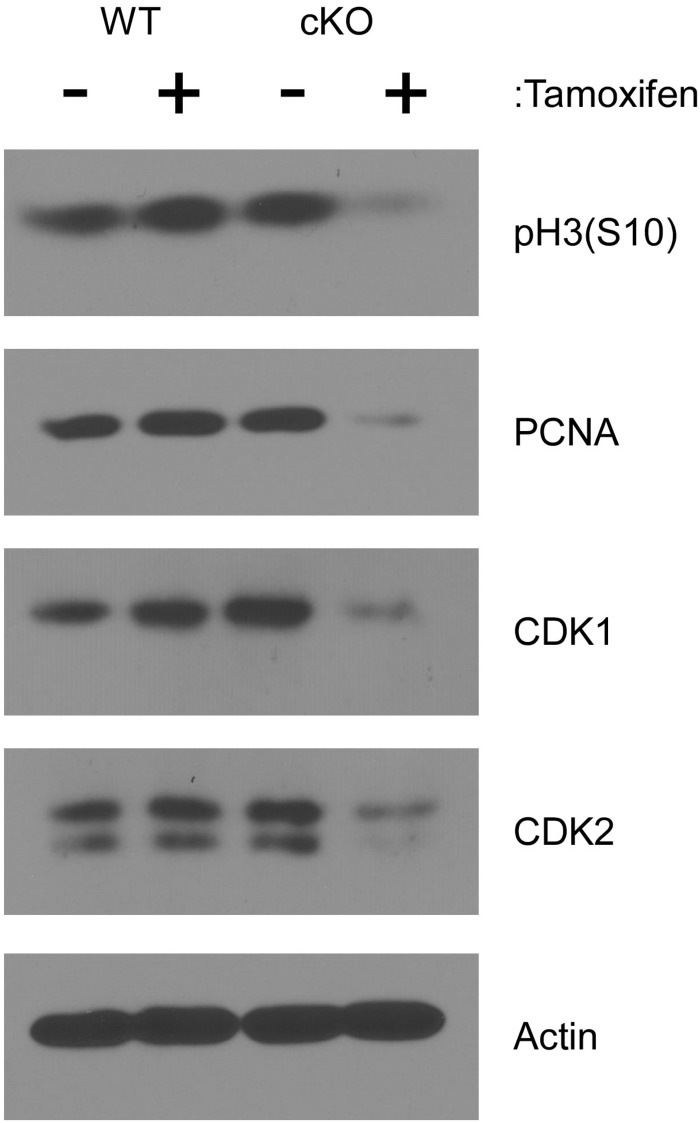
NKAP deficiency in hematopoietic progenitor cultures leads to cell cycle withdrawal. Hematopoietic progenitor cells as described in Figure 2 were either treated with tamoxifen for four days or left untreated. Cell lysates were then prepared from equal numbers of cells and analyzed by western blotting with the antibodies indicated. Actin was analyzed as a loading control. Data shown is representative of results obtained from three independent experiments initiated from separate pairs of mice.

To examine cell cycle progression at a molecular level, lysates from cultured cells were analyzed by western blot after four days of tamoxifen treatment. As shown in Figure 3, phosphorylation of histone H3 at serine 10 [pH3(S10)], which occurs specifically during mitosis (Bradbury, 1992), was substantially reduced in tamoxifen treated NKAP cKO cells, consistent with a reduced frequency of cell division. The level of PCNA was also reduced in NKAP deficient cells. PCNA, a component of the DNA replication machinery, is often downregulated as cells withdraw from the cell cycle and enter the G0 phase (Cheung and Rando, 2013). A substantial reduction in the levels of both CDK1 and CDK2 proteins was also observed in NKAP deficient cells (Figure 3). CDK1 is indispensable for cell cycle progression in many cell types (Aleem et al., 2005; Santamaria et al., 2007; Satyanarayana and Kaldis, 2009; Diril et al., 2012) and several studies have associated a reduction in CDK2 with senescence, or an irreversible withdrawal for the cell cycle (Freedman and Folkman, 2005; Zalzali et al., 2015).

**FIGURE 4:**
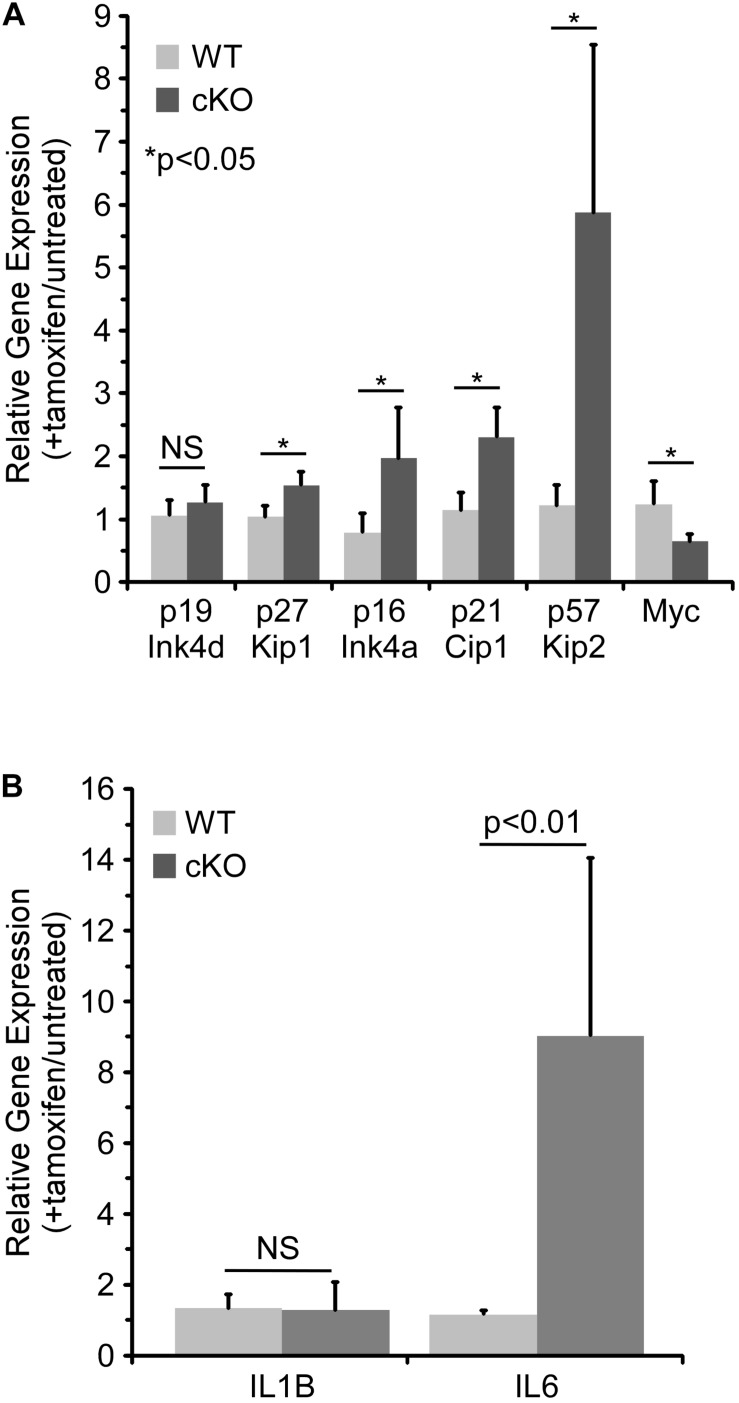
NKAP deficiency in hematopoietic progenitor cultures leads to upregulation of genes associated with cell cycle arrest and senescence. Hematopoietic progenitor cells as described in Figure 2 were either treated with tamoxifen for four days or left untreated. RNA was obtained from the cells and expression of the indicated genes determined by RT-PCR using Taqman gene expression assays (see section “Materials and Methods”). The data shown is averaged from four independent experiments initiated from separate pairs of mice. Gene expression relative to a normalizer was first calculated for each cDNA sample, and then the ratio of gene expression in tamoxifen treated cells relative to untreated cells of the same genotype determined. Note that for the WT cells, the data does not indicate any significant change upon tamoxifen treatment (relative expression of 1.0). **(A)** Expression of cell cycle regulators. **(B)** Expression of senescence associated cytokines.

As discussed above, p21 Cip1 has been shown to regulate cell cycle progression in HSCs (Cheng et al., 2000; Stier et al., 2003), as have two related CDKIs, p27 Kip1 and p57 Kip2 (Matsumoto et al., 2011; Zou et al., 2011). A member of the Ink family of CDKIs, p16 Ink4a, has also been shown to be a critical regulator of HSC proliferation (Janzen et al., 2006). Hence, the effect of NKAP deficiency on CDKI expression in cultured hematopoietic progenitor cells was examined by quantitative RT PCR (Figure 4A). As previously observed *in vivo* (Pajerowski et al., 2010), p21 Cip1 was significantly upregulated upon NKAP deletion, as was expression of p27 Kip1, p57 Kip2, and p16 Ink4a. Expression of Myc, a transcription factor that promotes proliferation in many cell types, was examined as well (Bretones et al., 2015). As expected, decreased Myc expression correlated with increased CDKI expression. Taken together, the changes in expression of numerous cell cycle regulators (Figures 3, 4A) demonstrate that NKAP deficiency leads to cell cycle withdrawal. The loss of CDK2 expression coupled with increased expression of p16 Ink4a and p21 Cip1 further indicate that NKAP deficiency may lead to senescence.

Senescence is an irreversible form of cell cycle withdrawal accompanied by a range of phenotypic changes (Evan and D’adda Di Fagagna, 2009; Childs et al., 2014) including increased production of certain cytokines [the “Senescence Associated Secretory Phenotype” (Coppe et al., 2008)]. For example, increased expression of IL6 and IL1β at the mRNA level was shown to occur during the development of senescence in multiple cell types (Kuilman et al., 2008). Thus, the effect of NKAP deficiency on the expression of several senescence associated cytokines was examined by quantitative RT PCR. While IL1β expression was unchanged, expression of IL6 increased substantially upon tamoxifen treatment of NKAP cKO cells (Figure 4B). Expression of CXCL1 and CSF2 were also examined, but they were below the limit of detection (not shown). The upregulation of IL6 in NKAP deficient cells is consistent with development of a senescent phenotype.

**FIGURE 5:**
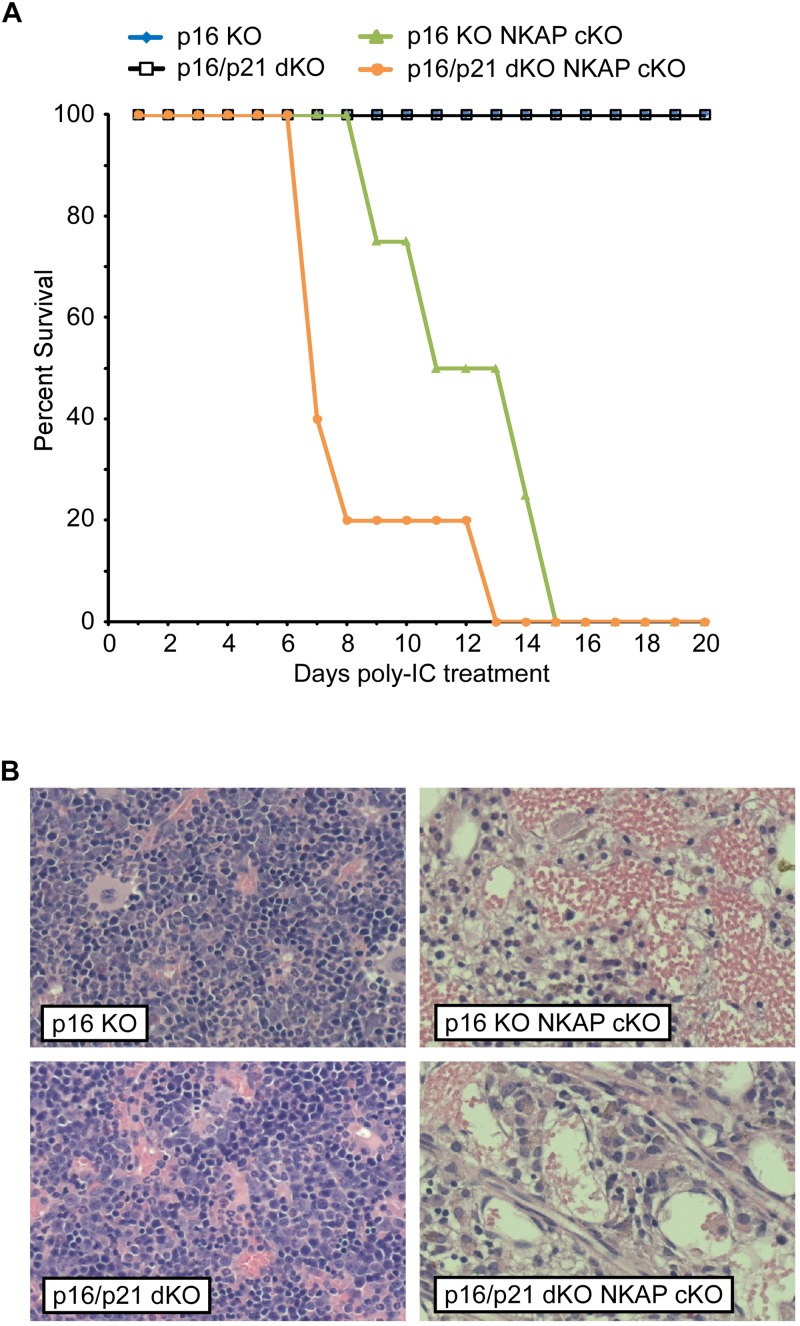
Lethality and hematopoietic failure resulting from NKAP deficiency is not abrogated when CDKI upregulation is impaired. **(A)** Survival was examined in 8 p16 KO, 4 p16 KO Mx1-cre NKAP cKO, 5 p16/p21 dKO, and 6 p16/p21 dKO Mx1-cre NKAP cKO mice (see section “Materials and Methods” for nomenclature). All mice were treated with poly-IC and monitored daily for up to 20 days. Lethality was recorded if the mice were found dead or required euthanasia due to severe morbidity. The percent of mice in each group surviving to the day indicated by the horizontal axis is shown on the vertical axis. **(B)** Femurs from poly-IC treated mice of the indicated genotypes were fixed in formalin at the end of the time course and paraffin embedded sections were then generated and stained with hematoxylin and eosin. Brightfield images were obtained and prepared as in Figure 1. Representative images are shown.

In addition to regulating cell cycle progression in general, p16 Ink4a and p21 Cip1 have been shown to be mediators of senescence specifically (Childs et al., 2014). As upregulation of both CDKIs was found to occur in NKAP deficient cells along with other indicators of senescence, it seemed possible that a deficiency in p16 Ink4a, alone or in combination with deficiency in p21 Cip1, would alter the effect of NKAP deficiency on hematopoiesis. Hence, p16 KO Mx1-cre NKAP cKO and p16/p21 dKO Mx1-cre NKAP cKO mice were analyzed. No lethality or changes in bone marrow cellularity resulted from poly-IC treatment of either p16 KO or p16/p21 dKO mice (Figure 5). However, poly-IC treatment in either p16 KO Mx1-cre NKAP cKO or p16/p21 dKO Mx1-cre NKAP cKO mice resulted in lethality within a similar time frame as in Mx1-cre NKAP cKO mice (Figure 5A), as well as similar effects on bone marrow cellularity (Figure 5B). To examine hematopoiesis in greater detail, peripheral blood counts were obtained (Table 1). No significant differences in blood counts between p16/p21 dKO and WT mice were observed, while, as described previously (Pajerowski et al., 2010), significant reductions were observed in all lineages in NKAP cKO mice upon Mx1-cre induction. Similar reductions were observed in p16/p21 dKO Mx1-cre NKAP cKO mice. Hence, combined deficiency in p16 Ink4a and p21 Cip1 did not compensate for the defect in hematopoiesis resulting from NKAP deficiency.

**TABLE 1:** The effect of NKAP deficiency on peripheral blood counts.

	**WT**	**NKAP cKO**	**p16/p21dKO**	**p16/p21dKONKAP cKO**
WBC (K/ml)	4.82 ± 0.48	1.03 ± 0.67	4.70 ± 0.72	0.51 ± 0.37
		*p* < 0.001	NS	*p* < 0.001
Neutrophils (K/ml)	1.19 ± 0.23	0.02 ± 0.01	1.60 ± 0.33	0.05 ± 0.05
		*p* < 0.001	NS	*p* < 0.001
Lymphocytes (K/ml)	3.48 ± 0.51	0.98 ± 0.66	2.99 ± 0.67	0.36 ± 0.31
		*p* = 0.001	NS	*p* < 0.001
Monocytes (K/ml)	0.15 ± 0.07	0.01 ± 0.01	0.08 ± 0.07	0.04 ± 0.06
		*p* = 0.004	NS	*p* = 0.055
Platelets (K/ml)	757 ± 228	39 ± 23	689 ± 120	57 ± 62
		*p* < 0.001	NS	*p* < 0.001
RBC (M/ml)	9.11 ± 0.43	2.92 ± 0.87	8.99 ± 0.45	2.33 ± 0.85
		*p* < 0.001	NS	*p* < 0.001
Hemoglobin (g/dL)	11.23 ± 0.50	3.08 ± 0.75	11.40 ± 0.81	2.88 ± 0.87
		*p* < 0.001	NS	*p* < 0.001
*n*	3	5	5	5

*Mice were treated with poly-IC to induce NKAP deletion and monitored daily for up to 20 days. Blood was obtained either at day 20 or at an earlier point if euthanasia was required due to severe morbidity. The genotype of the mice in each group is listed in the top row and the number per group in the bottom row. The cell types measured are listed in the left column. Each number is the average and standard deviation of the blood counts for a particular group. The *p*-values were determined by comparing each value to the same type of blood count in the WT group using an unpaired Student’s *t*-test. NS, not significant (*p* > 0.05).*

**FIGURE 6:**
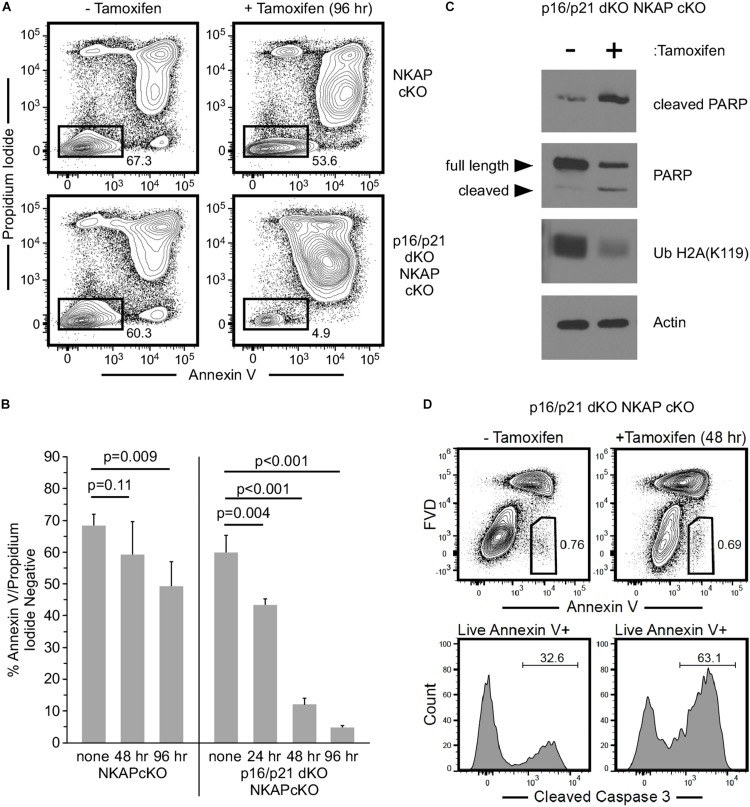
The consequence of NKAP deficiency is modified when CDKI upregulation is impaired in *ex vivo* cultures. Hematopoietic progenitor cells with a floxed NKAP gene and an ER-cre transgene (NKAP cKO) or cells also deficient in p16 Ink4a and p21 Cip1 (p16/p21 dKO NKAP cKO) were cultured *ex vivo* and treated with tamoxifen as indicated. Cells were then incubated with Annexin V and propidium iodide followed by flow cytometry to analyze apoptosis. **(A)** Representative flow cytometry data is shown for cells either left untreated or treated with tamoxifen for 96 h. ER-cre NKAP cKO cells are presented in the upper panels, and p16/p21 dKO ER-cre NKAP cKO are presented in the lower panels. As shown, the cells were divided into two populations, those labeled both by Annexin V and propidium iodide (apoptotic/dead), and those labeled by neither (viable). **(B)** The percent of viable cells was determined in cultures treated with tamoxifen for the indicated time periods or left untreated (none). Data shown is averaged from three independent experiments initiated from separate pairs of mice. **(C)** Lysates were generated from equal numbers of p16/p21 dKO ER-cre NKAP cKO cells either left untreated or treated with tamoxifen for 48 h and analyzed by western blotting. Lysates were examined with an antibody specific for the cleaved form of PARP, an antibody to total PARP (recognizing the full length and cleaved proteins), and an antibody to Ub H2A(K119). Actin was analyzed as a loading control. Data shown is representative of analysis performed on three separate cultures initiated from different mice. **(D)** p16/p21 dKO ER-cre NKAP cKO cells were either left untreated for treated with tamoxifen for 48 h and analyzed by flow cytometry for Annexin V binding in the presence of FVD (upper panels). Annexin V + FVD- live cells were examined for the presence of cleaved caspase-3 (lower panels). Data shown is representative of two separate cultures initiated from different mice.

To further examine the consequence of NKAP deficiency in cells incapable of either p16 Ink4a or p21 Cip1 expression, p16/p21 dKO ER-cre NKAP cKO cells were compared to ER-cre NKAP cKO cells in hematopoietic progenitor cultures. A rapid morphological change indicative of cell death in tamoxifen treated ER-cre NKAP cKO p16/p21 dKO cells was observed, suggesting that these cells were undergoing apoptosis. Cells were treated with tamoxifen for up to 4 days and Annexin V binding and permeability to propidium iodide were measured (Figures 6A,B). A small population of apoptotic cells was observed, and at similar levels, in both cultures prior to tamoxifen addition. In NKAP cKO cells, only a slight increase in this population occurred even four days after NKAP deletion was induced. However, a marked increase in apoptosis resulted from NKAP deletion in a p16/p21 KO background; over 95% of the cells were in the apoptotic population 4 days after tamoxifen treatment was initiated. Markers of apoptosis were also examined to determine whether this was the cause of cell death (Figure 6C). In tamoxifen treated ER-cre NKAP cKO p16/p21 dKO cells, the level of cleaved PARP was increased substantially and the level of uncleaved PARP was reduced relative to untreated cells, indicative of apoptosis (Lazebnik et al., 1994). In addition, NKAP deficiency resulted in the loss of ubiquitylation of histone H2A at lysine 119 [Ub H2A(K119)]. Caspase-dependent deubiquitination of histone H2A has been shown to occur along with PARP cleavage upon treatment of cells with a variety of apoptosis-inducing agents (Mimnaugh et al., 2001). In addition, NKAP deficiency also increased the percentage of cells with cleaved caspase-3 (Figure 6D). Consistent with the *in vivo* data above (Figure 5), these results show that preventing p16 Ink4a and p21 Cip1 expression is insufficient to abrogate the effect of NKAP deficiency in hematopoietic progenitors. However, it is clear that in the absence of p16 Ink4a and p21 Cip1, NKAP deficiency leads primarily to cell death rather than cell cycle withdrawal. The implications of this change of phenotype will be discussed below.

## Discussion

Prior work demonstrated that NKAP is essential for maintenance and survival of HSCs *in vivo*, and increased expression of p21 Cip1 and p19 Ink4d was also observed in NKAP deficient HSCs. NKAP was found to bind to the p21 Cip1 promoter (Pajerowski et al., 2010), to form a complex with two other transcriptional regulators, and to influence expression of Notch target genes (Pajerowski et al., 2009). Hence, it seemed plausible that the effect of NKAP deficiency on hematopoiesis could stem directly from impairment of the regulatory pathways controlling CDKI expression. However, as shown here, NKAP deficiency still resulted in hematopoietic failure in mice also deficient in p19 Ink4d, p21 Cip1, or p16 Ink4a (Figures 1, 5 and Table 1). Thus it is unlikely that NKAP functions solely to regulate expression of these genes.

To gain further insight into the role NKAP in HSCs, an *ex vivo* culture model was utilized. Taken together, the data indicates that NKAP deficiency primarily results in cell cycle withdrawal and the development of a senescent phenotype. NKAP deficient cells failed to expand in culture and exhibited a decrease in the level of pH3(S10), a marker of mitotic cells. Expression of Myc, a transcription factor that promotes proliferation (Bretones et al., 2015), was decreased and expression of several CDKI genes was increased. Further, the levels of PCNA, CDK1, and CDK2 proteins were markedly reduced in NKAP deficient cells (Figure 3). PCNA is required for DNA replication, and loss of its expression is a common indicator of cell cycle exit (Cheung and Rando, 2013). CDK1 and CDK2 both regulate cell cycle progression (Satyanarayana and Kaldis, 2009), for which CDK1 is thought to be indispensable (Aleem et al., 2005; Santamaria et al., 2007; Diril et al., 2012). Hence, the NKAP deficient cells analyzed here clearly lose the capacity to proliferate under conditions that are mitogenic for control cultures. Further, the increased p16 Ink4A and p21 Cip1 expression along with loss of CDK2 expression are indicative of senescence, a form of irreversible cell cycle withdrawal (Freedman and Folkman, 2005; Janzen et al., 2006; Hidalgo et al., 2012; Zalzali et al., 2015). Upregulation of the senescence associated cytokine IL6 (Coppe et al., 2008; Kuilman et al., 2008) was also observed in NKAP deficient cells. Thus, the loss of NKAP leads to senescence, and not simply cell cycle arrest.

Senescence is induced by a wide range of macromolecular damage and other forms of cellular stress (Evan and D’adda Di Fagagna, 2009; Childs et al., 2014). For example, HSC senescence is promoted by exposure of mice to ionizing radiation (Wang et al., 2006) and reactive oxygen species (Shao et al., 2011). Senescence is also associated with the accumulation of macromolecular damage during aging, and senescent HSCs detected in the bone marrow of aged mice displayed DNA damage foci and shortened telomeres (Flach et al., 2014; Wang et al., 2014). The CDKI p57, which was substantially upregulated in NKAP deficient cells, has been shown to limit proliferation in responses to oxidative and osmotic stress (Joaquin et al., 2012). Further, p21 upregulation occurs in response to genotoxic stress in HSCs (Asai et al., 2011). Apoptosis can be triggered by many of the same types of stress as senescence, and the two may be viewed as alternative cell fates influenced by the cellular context (Childs et al., 2014). Thus, it is notable that apoptosis rather than senescence occurred in NKAP deficient cells incapable of utilizing p16 Ink4a or p21 Cip1 driven pathways to withdraw from the cell cycle (Figure 6). Hence, it seems likely that NKAP deficiency causes one or more forms of stress that trigger senescence, or apoptosis when CDKI upregulation is impaired.

At present, the types of stress caused by the absence of NKAP are not certain. Recent work suggests that NKAP may ensure proper chromosome alignment during mitosis, and NKAP knockdown in HeLa cells resulted in the accumulation of chromosomal abnormalities (Li et al., 2016). NKAP associates with HDAC3, and HDAC3 deficiency has been reported to lead to increased DNA damage in fibroblasts and a block in DNA replication in hematopoietic cells (Bhaskara et al., 2008, 2010; Summers et al., 2013). To test this, phosphorylation of histone H2A. X was examined in our hematopoietic progenitor cultures. This chromatin modification occurs at sites of DNA damage and may accumulate at damaged telomeres during senescence (Nakamura et al., 2008). However, we have not observed any increase in phosphorylated histone H2A. X upon NKAP deletion (data not shown). NKAP also acts as a transcriptional repressor (Pajerowski et al., 2009) and the histone modifications mediated by HDAC3 have a well-established role in gene silencing. Additionally, NKAP has been shown to be involved in splicing and RNA processing (Burgute et al., 2014; Fica et al., 2019; Zhang et al., 2019). Hence, NKAP deficiency could lead to altered expression of multiple genes, perhaps along with abnormalities in other processes dependent on chromatin modification, resulting in a cumulative effect. While future work will be needed to further define the mechanisms involved, it is clear that NKAP has a critical function in cell survival and proliferation that is essential for the maintenance of a viable HSC pool.

## Data Availability Statement

The datasets generated for this study are available on request to the corresponding author.

## Ethics Statement

The animal study was reviewed and approved by Mayo Clinic Institutional Animal Care and Use Committee (IACUC).

## Author Contributions

MS and VS designed and performed the research, analyzed the data, and wrote the manuscript. ML performed the research and analyzed the data. JA, MC, and MN performed the research.

## Conflict of Interest

The authors declare that the research was conducted in the absence of any commercial or financial relationships that could be construed as a potential conflict of interest.

## Abbreviations

CDK: cyclin dependent kinase
CDKI: cyclin dependent kinase inhibitor
cKO: conditional knockout
dKO: double knockout
ER-cre: estrogen receptor cre
FVD: fixable viability dye
HSC: hematopoietic stem cell
KO: knockout
PARP: poly (ADP-ribose)polymerase
PCNA: proliferating cell nuclear antigen
pH3(S10): serine 10 phosphorylated histone H3
Ub H2A(K119): lysine 119 ubiquitylated histone H2A
WT: wild type.
